# Comparison of the Level of Boron Concentrations in Black Teas with Fruit Teas Available on the Polish Market

**DOI:** 10.1155/2014/898425

**Published:** 2014-10-14

**Authors:** Anetta Zioła-Frankowska, Marcin Frankowski, Karel Novotny, Viktor Kanicky

**Affiliations:** ^1^Department of Water and Soil Analysis, Faculty of Chemistry, Adam Mickiewicz University in Poznań, Umultowska 89b, 61-614 Poznań, Poland; ^2^Department of Chemistry, Faculty of Science, Masaryk University, 611 37 Brno, Czech Republic

## Abstract

The determination of boron by inductively coupled plasma-atomic emission spectrometry has been carried in water-soluble and acid soluble (total content) fractions of 36 samples of traditional black tea and fruit brew. The estimation of the impact of the type of tea on the concentration of boron in water-soluble and acid extracts and potential human health risk from the daily intake of boron was carried out in this study. The levels of boron differed significantly in black and fruit tea types. The mean total content of boron ranged from 8.31 to 18.40 mg/kg in black teas, from 12.85 to 15.13 mg/kg in black tea with fruit flavor, and from 12.09 to 22.77 mg/kg in fruit brews. The degree of extraction of boron in black tea ranged from 8% to 27% and for fruit tea from 17% to 69%. In addition, the values below 25% were of black teas with fruit flavors. The daily intake of B from tea infusions (three cups/day) is still within the average daily intake except for some of the fruit brews which exceed acceptable regulations of the daily intake of total boron by humans. Hence, it may not produce any health risks for human consumption, if other sources of metal contaminated food are not taken at the same time.

## 1. Introduction

Boron is an essential element affecting the growth and development of plants [1, 2]. In the form of boric acid or borates it is necessary for the growth of land plants, especially vascular plants, and thus indirectly essential for the continuation of animal life. However, in large amounts the above compounds are toxic to organisms in general, including plants [3]. The toxicity symptoms of boron can vary from the necrosis of some plant organs to the death of the whole plant depending on the extent and severity of toxicity [4].

The fundamental importance of boron to animals and humans has not been identified precisely, although some evidence strongly indicates that boron is probably an essential micronutrient [2, 4, 5]. The presence of boron has been demonstrated to favorably affect the function and composition of several body compartments, such as the brain, the skeleton, and also the immune system in humans [2, 6]. Too high concentration of boron in human body may result in nausea, vomiting, diarrhea, and lethargy. The Word Health Organization has announced that a safe range of boron intake for adults is 1–13 mg/day [6].

It should be underlined that the existing evidence from plant, bacteria, animal, and human experiments shows that boron is a dynamic trace element affecting an exceptionally large number of seemingly unrelated biological functions [7]. That is why the control of boron levels in biological samples, especially those related to human nutrition, is needed [8]. It has been stated that food is a primary source of boron that is ingested by humans. Starting in the 1870s, for about 50 years, the addition of borates was one of the best methods of preserving food products. The research by Wiley [9], which presented the evidence of the negative effects of boric acid consumption in doses greater than 0.5 g per day for 50 days, contributed to the fact that borates and boric acid were essentially forbidden as food preservatives throughout the world [5]. Taking into account that excess of boron is toxic to human health, its analysis in food is becoming increasingly important [8].

The most popular nonalcoholic beverage consumed by about half of the world's population is tea* (Camellia sinensis* L.) prepared from steamed and dried leaves [10]. Tea can be classified into six basic categories: white, green, yellow, oolong, black, and pu-erh [11, 12]. Moreover, the types of tea are distinguished on the basis of the degree of fermentation, on which the final composition of tea, including its aroma, colour, and taste, is also dependent [13]. It is well known that tea contains many different compounds, including minerals and trace elements. The elements included in a brew of tea are differentially extracted into infusions, so the prepared beverage can be a reliable dietary source of essential major, minor, and trace elements, including boron [12, 14].

Currently used methods of boron determinations are spectrophotometric methods based on the creation of colored complex of boron with a suitable reagent, which have low precision and numerous interferences [15]. In the case of atomic absorption methods, despite the better sensitivity and lower detection limits they are burdened with interference and memory effect and the loss of boron in the case of not applying the appropriate modifier (GF-AAS). However, the use of ICP-AES methods provides higher sensitivity, lower detection limits, and less chemical interferences and is less time consuming [16, 17]. The ICP-AES method was used for the determination of boron in many food products, for example, in honey [18], peanut kernels [19], nuts and seeds [20], onions [21], herb samples [22], and liquid nutritional foods [23].

The aims of this study were to determine the total content of boron in samples of teas available on the Polish market, to mark the concentration of boron in the water-soluble fraction of tea samples, to establish the impact of tea type on the boron concentration in water and acid extracts and to estimate the daily intake of boron on the basis of boron in a cup of tea, and to determine the potential human health risk.

## 2. Material and Methods

### 2.1. Sample of Tea

The concentrations of boron were determined in commercially available teas in tea bags. The characteristics of analyzed teas are listed in Table 1.

### 2.2. Sample Tea Preparation

Two different procedures were applied in order to prepare the tea samples, including total decomposition and one single step extraction with water as an extractant. The total decomposition was carried out in a microwave system Mars Xpress (CEM, USA) based on the modified EPA 3051 method [24].

In the case of tea infusion as a water extract, 10 g of each sample of tea was accurately weighed into a polypropylene flask and extracted with 100 mL double distilled water DDW by mixing for 1 hour in 80°C. After the extraction, the contents of the flask were filtered through a filter and then boron determination was done by the ICP-AES analytical technique, without acidification of the water tea extract.

For the water extract of boron in tea, the sample pH was determined. The pH (H_2_O) was determined using the Orion 5-star Plus meter (Thermo, USA) with a Single Pore pH electrode (Hamilton, USA).

### 2.3. Analytical Method/Apparatus

The measurements of boron in tea and fruit brew samples were carried out by the ICP-AES with radial torch equipped with argon saturation assembly (iCAP 6500 duo, Thermo, UK). The emission lines of boron used were B 208.959 nm, B 249.678 nm, and B 249.773 nm. The operating condition was as follows: plasma power supply 1100 kW, observation height 15 mm, plasma gas flow 12 L/min, sheet gas flow 0 L/min, nebulizer gas flow 0.75 L/min, photomultiplier voltage 800 V, sample uptake rate 1.0 mL/L, integration time 1.0 s, and sample time delay 30 s. The instruments were calibrated for boron before analysis. The determination of boron was performed in 3 replications, and the %RSD did not exceed 5%.

To check the analytical procedure of the determination of boron in water-soluble and acid soluble (total content) fractions, the determination of reference material NIST SRM 1515 (National Institute of Standards and Technology, USA) was performed. The certified value for the reference material SRM 1515 was determined by ICP-AES technique. The reference material was analyzed during six repetitions by ICP-AES technique. The mean value, standard deviation, and method recovery [%] were calculated. The certified value for SRM 1515 (Apple Leaves) for boron is 27 ± 2 mg/kg. The determined value for acid soluble fraction (mineralization by modified EPA 3051 method) was 27.68 ± 1.18 mg/kg, with recovery 102.5%, and for water-soluble fractions the obtained value was 15.19 ± 0.494 mg/kg, with recovery 56.26%.

## 3. Results and Discussion

### 3.1. Total Concentration of Boron in Teas

**Figure 1:**
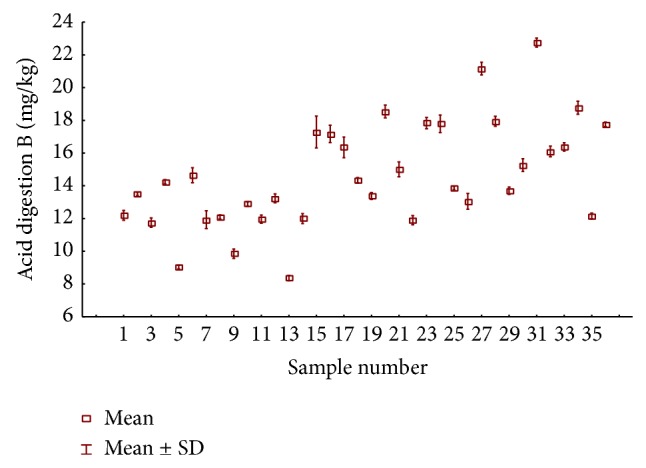
The concentration of boron mg/kg in tea and fruit brew samples by acid extraction.

The results of total content of boron in black and fruit tea are presented in Figure 1. The tea samples were distinguished as black teas (samples 1–24), black teas with fruit flavor (samples 25, 26, and 30), and fruit teas as typical fruit brews (samples 27–29 and 31–36). The highest total content of boron in black tea was 18.4 mg/kg (sample 20), and the lowest was 8.31 mg/kg (sample 13). For black tea with fruit flavor, the boron concentration amounted to 12.9–15.1 mg/kg. In fruit brew samples, boron content was determined at a higher level (from 12.1 to 22.8 mg/kg). However, the average total concentrations of boron were as follows: fruit brew 17.4> black tea with fruit flavors 13.9> black tea 13.5 mg/kg. It should also be noted that, in the group classified as fruit tea, only samples 25, 26, and 30 contained tea leaves; the remaining samples consisted of a mix of dried fruit. Thus, the total concentrations of boron indicated were similar to those obtained for typical black teas. The boron concentrations determined by Krejčová and Černohorský [16] in black tea samples were on the level of mean values determined by us, while the boron concentrations in fruit brews were also higher in comparison with black teas. Özcan et al. [26] studied the mineral content of herbs and teas, determining boron in black tea samples at the range of 6.8–24.6 mg/kg. Malik et al. [27] determined total content of boron in black tea at the range of 14.2 to 21.1 mg/kg, while in the rooibos (*Aspalathus linearis*) plants (green and red species) the element content was determined at range from 13.0–20.3 mg/kg, which is in accordance with the concentrations obtained for fruit brew samples.

In turn, in* Hibiscus sabdariffa *L. (Malvaceae), commonly known as Roselle, which is one of the most economically important herbal tea plant species [28] determined the content of boron at the range of 19.18 to 27.44 mg/kg. Our results for the total content of boron in fruit teas which contain hibiscus are in accordance with those of [28]. It is worthy to say that in some herbal teas the concentration of boron is much higher than in typical black or fruit teas. For example, the determined content of boron in sage (*Salvia fruticosa L.*) was 46.7 mg/kg, in coriander (*Coriandrum sativum L.*) it was 41.8 by Özcan et al. [26], for nettle (*Urtica dioica*) it was 61.00 mg/kg by Pytlakowska et al. [29], and for* Ginkgo biloba L.* (Ginkgoaceae) it was 167.8 mg/kg by Stefanovits-Bányai et al. [30]. Summing up the content of boron varied between 8.31 and 22.8 mg/kg in the analyzed tea samples. According to the literature, most of the investigated foodstuffs contained less than 1 mg/kg of B; for example, 0.074 mg/kg was found in chicken, 0.82 mg/kg in dried shrimp, and 0.55 mg/kg in rice [8].

### 3.2. Water Soluble Fraction of Boron in Tea Samples

**Figure 2:**
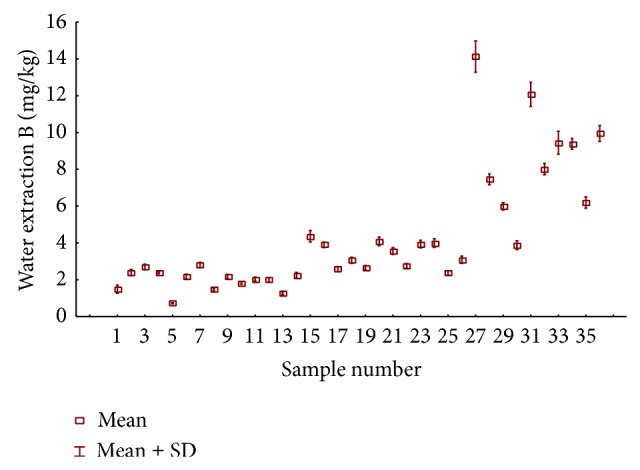
The concentration of boron mg/kg in tea and fruit brew samples by water extraction.

Boron was determined in water-soluble fraction in the same type of tea samples as for the total content determination (Figure 2). The highest concentration of boron in water-soluble fraction in black teas was determined in sample 15 (4.49 mg/kg) and the lowest in sample 5 (0.72 mg/kg). It was observed that the concentrations of boron in water-soluble fraction of fruit teas are several fold higher in comparison with the boron concentrations found in black teas. The highest concentration of boron in water soluble fraction in fruit brew samples was 14.48 mg/kg and it was on the level of total boron concentrations determined in black teas. Krejčová and Černohorský [16] determined boron in water-soluble extracts within the range from 3.21 to 9.25 mg/kg in black teas and from 2.71 to 27.7 mg/kg in fruit teas.

It should be also noted that, in the group of teas classified as fruit teas, only samples 25, 26, and 30 contained tea leaves, while the remaining samples contained a mix of dried fruit (Table 1). That is why the boron concentrations, both total and those determined in water-soluble fraction, are at a similar level as the concentrations in black teas. Higher concentrations of boron in water-soluble fraction of fruit tea samples may be linked to the fact that these samples consisted of dried fruit, which contain higher concentrations of boron than tea leaves.

Extraction efficiency was calculated as a ratio of boron concentration in the water-soluble fraction to the element concentration in tea samples obtained after the complete decomposition in the microwave system (Table 1). The extraction level of boron for black teas ranged between 8% and 27%, and for fruit tea it was from 17% to 69%. In addition, the values lower than 25% were found for black teas with fruit flavours (samples 25, 26, and 30). It should be noted that a very high level of boron extraction was obtained from tea samples containing a mix of dried fruit, using the least aggressive extractant that is water.

According to the extraction efficiencies boron has been classified as the element moderately extractable within the range of 20 to 55%, which is in accordance with results obtained by us [12]. Also, the high levels of boron extraction obtained in the samples of dried fruit teas may be the evidence of another mechanism of binding boron in fruit in comparison with black tea leaves, especially considering the fact that fruits contain numerous polyphenols, which consist, among others, of mono sugars and other compounds containing cis-hydroxyl groups which can form complexes with boron (e.g., as boron-diol complexes with mannitol or sorbitol) [1, 4, 5, 7].

Besides, the obtained results of pH value of water-soluble fraction of tea samples (Table 1) indicate that a lower pH in the case of fruit brew favors higher concentrations of boron in the water fraction, and it can be connected with greater degree of leaching of boron to the infusion. In the case of black teas it was observed that at higher pH values determined boron concentration was much lower than those marked in the fruit teas.

### 3.3. Concentration of Boron in Cup of Tea

According to current studies, regular consumption of tea can be significantly associated with the daily dietary uptake of certain elements [28]. Taking into account that the quantitative estimate of boron is of particular importance in order to assure its nutritional integrity and, in consequence, the human health. That is why the potential concentration of boron in a cup of tea (in our case 200 mL) was calculated, based on the results determined for water-soluble fraction of tea samples (Table 1).

The results indicate that the boron concentration varies between 0.29–1.45 mg/per a cup of black tea, 0.73–1.19 mg/per a cup of black tea with fruit flavours, and 2.52–7.24 mg/per a cup of fruit brew. According to the literature, the main source of dietary B is beverages (31%); the amount contained in hibiscus (up to 5.579 mg/L) makes it possibly one of the top B contributors for humans [28]. Also in our findings the highest concentration of boron was found in tea containing hibiscus (sample 27). One should remember that typical fruit teas are produced from dried fruit—dried raspberries, cherries, blackberries, and cranberries. These are often mixed with hibiscus flower (which can be found in almost every fruit tea), as well as with fruit or wild rose petals and with the addition of herbs. For the above reason, such beverage is not real tea, but rather an infusion, as it does not contain tea leaves (*Camellia sinensis*). Moreover, the boron concentrations determined by Özcan et al. [26], in the infusion of teas, were much lower and amounted to 0.035 mg B/100 mL for the infusion of black tea and 0.214 mg B/100 mL for* Matricaria chamomilla*. Özcan et al. [26] marked by an order of magnitude lower boron concentration in the infusion of black tea compared to the herbs. This finding is in accordance with our results, where determined concentrations in black tea were also much lower in comparison with dose of fruit brew sample.

It should be noted that the content of metals, including boron, in teas and infusions may depend on physiological properties or structures of ingredients of teas, levels of phytochelatin phenolic, other mineral-binding components, and the pH of the water used in tea preparation and on the solubility of metals and other mineral elements in hot water [10, 12, 31].

The obtained results of concentration of boron in cup of tea were compared with upper limit for boron intake which is given in Table 2 [25].

The presented ranges of concentrations for the daily boron intake vary considerably depending on the research centre which conducted the determinations. Nevertheless, the determined concentrations of boron in a cup of tea are relatively high, especially for fruit teas.

In order to estimate the potential health risk associated with the consumption of boron, the total amount of boron tea infusions was calculated for the daily intake. The calculated amounts are based on the concentrations of boron in tea infusion as a cup of tea and the assumption that the average consumption of tea for a single person is three cups a day (each with 200 mL per cup) with one single tea bag and are presented in Table 1.

Taking into account the fact that we drink more than one cup of tea on average and assuming the determined boron concentrations in a cup of tea, only drinking tea itself covers and for certain tea samples (fruit brew) even exceeds safety regulations of the daily intake of boron by humans. Murray and Schlekat [32] analysed the defined by the other researchers tolerable daily boron intake recommendations amounting from 10 to 24 and 12 to 28 mg B/day for 60 kg women and 70 kg men, respectively. The safe boron intake level for human was estimated by seven different research centers, which were based on the same, single study in rats. That is why the ranges of boron concentration in the daily human diet assumed as not harmful should be treated as arbitrary and not reflecting the actual boron concentrations consumed.

## 4. Conclusions

Based upon the obtained results of this study, the following conclusions can be made.

Fruit brew samples (both acid and water soluble fraction) have boron concentrations greater than the concentration obtained in typical black teas. As a result, teas, especially fruit, were found to be significant sources of boron. The average concentration of boron in a cup of tea was about 4.5-fold higher in fruit teas than in black teas. Fruit teas containing hibiscus were marked by the highest concentrations of boron in comparison with the other investigated teas. Regular daily intake of tea (3 cups), especially ones with dried fruit, covers the daily demand for boron in humans.

It was found that, with respect to acceptable daily intake boron (three cups/day) in daily dietary standards, the infusion of black tea samples analyzed in the present study was found to be safe for human consumption. On the other hand some of the fruit brews exceed acceptable regulations of the daily intake of total boron by humans. However it may not produce any health risks for human consumption, if other sources of metal contaminated food are not taken at the same time.

That is why it is important to determine the concentration of boron in food, beverages, dietary supplements, and care products to avoid overconsumption and accumulation of boron over a long period of use.

## Figures and Tables

**Table 1:** Information on the tea types, ingredients, origins for the analyzed tea samples, and the result of concentration of boron in cup of tea, fruit brew samples, calculated amounts of boron in tea infusion as three cups a day (200 mL/cup), and calculated percentage of the water soluble fraction of boron and the pH value for water extracts.

Sample	Type of tea	Composition of tea and ingredients	Origin	Tea weight per bag [g]	Concentration of boron in cup (200 mL) of tea and fruit brew [mg/200 mL]	Calculated amounts of boron in tea infusion as three cups a day (200 mL/cup)	Calculated percentage of the water soluble fraction of boron [%]	pH value for water extracts
1	Black	100% tea leaves	Kenya, India (Assam)	3.00	0.877 ± 0.115	2.630 ± 0.345	12.09	5.071
2	Black	100% tea leaves	Africa, India (Assam)	2.00	0.984 ± 0.057	2.951 ± 0.172	18.19	4.948
3	Black	Tea leaves with bergamot flavored. Lemon aroma. Gluten	India, Kenya, Ceylon	1.50	0.829 ± 0.041	2.487 ± 0.122	23.74	4.811
4	Black	100% tea leaves	India (Assam)	2.00	0.955 ± 0.020	2.866 ± 0.059	16.78	5.288
5	Black	100% tea leaves	China (Yunnan)	2.00	0.289 ± 0.009	0.868 ± 0.027	7.983	4.973
6	Black	100% tea leaves	Ceylon	2.00	0.893 ± 0.046	2.680 ± 0.138	15.45	6.27
7	Black	100% tea leaves. Bergamot flavor	NA^a^	2.00	1.144 ± 0.050	3.431 ± 0.150	24.21	4.653
8	Black	100% tea leaves. Bergamot flavor	Ceylon (region of Dimbula)	1.50	0.446 ± 0.017	1.338 ± 0.051	12.33	4.782
9	Black	100% tea leaves	Himalayas	1.40	0.621 ± 0.028	1.862 ± 0.083	22.79	4.567
10	Black	100% tea leaves	Sri Lanka	2.00	0.742 ± 0.024	2.226 ± 0.073	14.35	6.425
11	Black	100% tea leaves	India (Assam)	1.75	0.726 ± 0.034	2.178 ± 0.101	17.43	5.577
12	Black	100% tea leaves. Lemon peel	NA^a^	1.70	0.692 ± 0.031	2.077 ± 0.094	15.47	4.526
13	Black	NA^a^	NA^a^	1.75	0.454 ± 0.023	1.361 ± 0.070	15.61	4.903
14	Black	NA^a^	NA^a^	1.50	0.691 ± 0.040	2.073 ± 0.120	19.39	5.955
15	Black	NA^a^	NA^a^	1.50	1.346 ± 0.080	4.037 ± 0.241	26.56	4.882
16	Black	100% tea leaves	India (Assam)	1.30	1.029 ± 0.034	3.086 ± 0.101	23.24	6.887
17	Black	100% tea leaves	Sri Lanka	1.75	0.921 ± 0.040	2.763 ± 0.121	16.32	4.737
18	Black	100% tea Leaves. Bergamot flavor	Indonesia, Kenya, Sri Lanka	1.40	1.091 ± 0.051	3.274 ± 0.153	21.80	4.62
19	Black	NA^a^	NA^a^	1.40	0.757 ± 0.028	2.270 ± 0.085	20.30	4.866
20	Black	NA^a^	NA^a^	2.00	1.171 ± 0.058	3.513 ± 0.174	22.73	4.505
21	Black	NA^a^	NA^a^	1.50	1.454 ± 0.064	4.363 ± 0.192	24.53	4.582
22	Black	NA^a^	NA^a^	1.50	0.953 ± 0.375	2.859 ± 1.126	26.95	5.829
23	Black	NA^a^	NA^a^	1.40	1.208 ± 0.054	3.624 ± 0.163	22.73	5.266
24	Black	NA^a^	NA^a^	1.40	1.145 ± 0.057	3.436 ± 0.171	23.26	5.047
25	Fruit	Tea leaves with strawberry flavor	Sri Lanka	1.50	0.725 ± 0.030	2.176 ± 0.090	17.41	4.765
26	Fruit	Tea leaves with strawberry flavor	NA^a^	1.40	0.887 ± 0.048	2.660 ± 0.144	24.65	4.578
27	Fruit brew	Hibiscus. Rosehip. Apple. Orange peel. Raspberry. Elderberry fruit	NA^a^	2.50	7.241 ± 0.368	21.72 ± 1.105	68.56	2.702
28	Fruit brew	Hibiscus. Chokeberry. Apple. Blackberry leaf. Rosehip. Flavors. Lemon peel. Orange Peel. Black Currant. Strawberry fruit 0.1%	NA^a^	2.00	3.031 ± 0.101	9.094 ± 0.302	42.50	2.641
29	Fruit brew	Hibiscus Flower. Wild rose 20%. Black currant 11%. Aronia 10%. Apple 9.3%. Orange peel 2%	NA^a^	2.30	2.793 ± 0.081	8.379 ± 0.243	44.61	2.741
30	Fruit	Tea leaves. Ginger	Ceylon	1.50	1.193 ± 0.062	3.580 ± 0.185	26.29	4.533
31	Fruit brew	Hibiscus. Apple. Blackberry leaf. Flavors. Rosehip fruit cranberries 1% citric acid. Acidity regulator-fruit raspberry 0.3%	NA^a^	2.00	4.940 ± 0.226	14.82 ± 0.679	54.24	2.560
32	Fruit brew	Wild rose 55%. Hibiscus flower 30%. Raspberry flavor. Raspberry fruit 3%. Citric acid. Acidity regulator	NA^a^	2.00	3.258 ± 0.107	9.775 ± 0.321	50.85	2.656
33	Fruit brew	Rosehip. Hibiscus flower. Cherry flavor. Granulated yogurt 1%	NA^a^	3.00	5.828 ± 0.327	17.48 ± 0.980	59.37	2.958
34	Fruit brew	Hibiscus flower. Apple, blackcurrant 7%. Raspberry 6%. Licorice 4%. Blackberry 4%. Blueberry 4%. Dried juice of 2%. Malic acid. Acidity regulator	NA^a^	2.00	3.799 ± 0.102	11.40 ± 0.306	51.00	2.644
35	Fruit brew	Raspberry 40%. Chokeberry. Elderberry. Apple. Flavoring. Citric acid	NA^a^	2.00	2.524 ± 0.106	7.573 ± 0.319	52.20	2.809
36	Fruit brew	Wild rose 56%, hibiscus. Raspberry flavor. Raspberry (1% fruit. Maltodextrin-from starch)	NA^a^	2.00	4.052 ± 0.150	12.16 ± 0.450	57.07	2.804

^a^Data not available.

**Table 2:** Reference data of daily intake of boron for human acquired from literature [25].

Name of organizations	Acceptable/safe levels of daily intake of boron by human [mg/day]	Type of sample
Scientific Panel on Dietetic Products, Nutrition and Allergies	3–10	Food, dietary supplements, and drinking water
NCEA (National Center for Environmental Assessment of the Environmental Protection Agency (EPA)	14	Drinking water
EFSA (European Food Safety Authority)	10 (adults) 3 (1–3 yrs); 4 (4–6 yrs); 5 (7–10 yrs), 7 (11–14 yrs), 9 (15–17 yrs)	Drinking water
EVGM (Expert Group on Vitamins and Minerals)	10	Food, dietary supplements, and drinking water
FNB (US Food and Nutrition Board)	20	Food, dietary supplements, and drinking water
WHO (World Health Organization)	1–13	Food, dietary supplements, and drinking water
Tolerable intake (TI) by WHO (World Health Organization)	28 mg/day for a 70 kg adult	Food, dietary supplements, and drinking water
